# Development of Novel Continuous and Interval Exercise Programs by Applying the FITT-VP Principle in Dogs

**DOI:** 10.1155/2020/3029591

**Published:** 2020-04-13

**Authors:** H. S. Lee, S. H. Lee, J. W. Kim, Y. S. Lee, B. C. Lee, H. J. Oh, J. H. Kim

**Affiliations:** ^1^Department of Physical Education, College of Performing Art and Sport, Hanyang University, 222 Wangsimni-ro, Seongdong-gu, Seoul 04763, Republic of Korea; ^2^Department of Theriogenology and Biotechnology, College of Veterinary Medicine, Seoul National University, 1 Gwanak-ro, Gwanak-gu, Seoul 08826, Republic of Korea

## Abstract

Although proper exercise training induces positive physiological effects, improper exercise can lead to injury, fatigue, and poor performance. The frequency, intensity, time/duration, type, volume, and progression (FITT-VP) are the essential components of exercise training to maintain or improve physical fitness and health. The purpose of this study was to develop specific exercise programs by applying the FITT-VP principle and to examine the effects on heart rate (HR) and hematological and biochemical parameters in dogs. The healthy male Beagles (*n* = 4) included in this study performed continuous and interval exercises, comprising 12 protocols. The HR monitoring elicited an affirmative response to activities but varied depending on the protocols. The hematologic parameters (e.g., red blood cell count, white blood cell count, hemoglobin, mean corpuscular hemoglobin, and mean corpuscular hemoglobin concentration) were within the reference ranges both before and after exercise. The creatine kinase level significantly increased, and the cholesterol level decreased after exercises. In conclusion, the continuous and interval exercise program elicits an appropriate HR reaction, has no adverse effects on the serum parameters, and provides valuable insight for healthcare in dogs.

## 1. Introduction

It is known from many studies that appropriate and effective levels of exercise bring about improvement in physical activity [1] and cognitive and mental functions [2] and have social benefits [3]. However, inappropriate or ineffective exercise can cause injury, fatigue, and poor performance [4, 5]. In humans, systematic exercise programs under the principle of frequency, intensity, time/duration, type, volume, and progression (FITT-VP) were established in the field of sports and exercise science [6, 7]. Studies on aerobic fitness, musculoskeletal disease, and motor functions have been actively reported [8]. The principles of FITT-VP are essential for designing effective exercise programs [9–11]. The FITT-VP principle is most commonly applied to continuous exercise and interval exercise programs [12], but the advantages and efficiency of these two exercise programs are controversial [13, 14]. Traditionally, continuous exercise, characterized by repeated isotonic contraction of the large skeletal muscle groups, is the most common type of exercise known to improve body composition, physical capacity, and overall health [12, 15]. On the other hand, interval exercise involves varying exercise intensities within a single exercise session. Short-term (≤1 month) interval exercise improved the cardiorespiratory fitness and cardiometabolic biomarkers (i.e., blood lipoproteins, glucose, and interleukin-6) compared with continuous single-intensity exercise in healthy people [16, 17]. Although continuous and interval programs comprise different exercise components and outcomes, they are the most recommended types of exercise for improving performance and health with positive impacts on the physiological parameters in humans [18, 19].

Dogs and humans are known to have similar physiological metabolism and anatomical structures [20, 21]. In dogs, exercise can cause changes in many physiological parameters. In the studies using Labrador retrievers and Beagles, the incremental treadmill exercise-induced changes in the acute blood lactate, heart rate (HR) [22], and body temperature were reported [23]. Additionally, low-to-high intensity (6% to 20% slope) treadmill exercise considerably affected the physiologic regulators (i.e., cortisol, glucose, and norepinephrine) of both the peripheral and central neuroendocrine systems [24]. In untrained Beagles, moderate treadmill exercise led to positive changes in lactate, glucose, HR, rectal temperature, and hematological parameters [25]. In healthy military dogs, acute exercise decreased gastric motility, caused cardiovascular changes, and altered biochemical parameters [26]. Although previous studies have evaluated the effects of exercises on dogs, no studies have established systematic exercise programs by applying the FITT-VP principle. Instead, only simple and repetitive exercise programs were used (i.e., low frequency, short-cycle, and same or similar intensity of exercise), and such a simple form of exercise could limit the improvement in health and physical functions. Therefore, we hypothesized that dog-specific exercise programs applying the human FITT-VP principle would overcome these limitations. To establish the dog-specific exercise programs, we created and evaluated the two most representative exercise programs used in humans: continuous exercise and interval exercise [12].

In order to evaluate the programs in this study, we checked the HR, as it is an essential indicator to assess the effects of exercise in humans [27, 28]. It is a noninvasive method, which can indicate the clinical status of the cardiac function and presence/absence of cardiovascular diseases in exercise studies [23]. Hematological and biochemical analyses of the blood can assess the physiological and pathological conditions in dogs [25, 29]. These indicators were used to assess whether there was any detrimental effect of the dog-specific exercise programs. Therefore, the purpose of this study was to develop specific continuous and interval exercise programs for dogs using the principle of FITT-VP, as well as investigate the physiological changes caused by these programs by analyzing HR and hematological and biochemical parameters.

## 2. Materials and Methods

### 2.1. Animals

This study included four 2-year-old healthy male Beagles, and their baseline information is shown in Table 1. All Beagles used in this study were cared for as per the recommendations described in “the Guide for the Care and Use of Laboratory Animals” of the Institutional Animal Care and Use Committee (IACUC) of Seoul National University (SNU-180731-2). All Beagles were subjected to the same dietary and resting conditions. Before the start of the experiments, a veterinarian conducted blood tests, serum tests, and body composition tests for the participants. The dogs were housed in an environment of 12 hr (07:00–19:00) bright light and 12 hr (19:00–07:00) darkness. The temperature of the housing was 22-23°C, with 50–60% humidity. Aside from the exercise, all dogs had a >30 min free outdoor walk every day for fresh air and were kept in separate cages (775 × 960 × 900 cm) with soft rubber flooring, which was cleaned daily. Meals were served twice a day (09:00, 17:00), and freshwater was provided *ad libitum*.

### 2.2. Study Design

All Beagles received two weeks of adaptive training to familiarize themselves with the researcher, laboratory environment, and training procedures and equipment before the main experiment. The Beagles performed exercise on an automated treadmill (EGOJIN XG-V6E, Gyeonggi-do, Korea) wearing a safety belt applied to the chest. The tester observed the dog's movement and confirmed safety throughout the experiment. None of the Beagles attempted to escape the treadmill during the experiment, but the researchers applauded and called out their names to encourage the dogs to concentrate on the exercise when they showed signs of distraction. The rectal temperature was measured before and after adaptive training using a digital thermometer.

#### 2.2.1. Continuous Exercise

Continuous exercise, a form of physical training that involves a workout without resting intervals, was provided three times a week for four weeks and consisted of 12 treadmill protocols. All protocols were divided into six working (W) stages, labeled W1, W2, W3, W4, W5, and W6. In protocols 1 to 6 of continuous exercise, six W stages in one protocol progressed at the same grade and speed, and the intensity (grade, speed, and time) of the exercise was gradually increased as the protocol proceeded. In protocols 7 to 12, the grade and speed were gradually increased at six W stages within one protocol, and the grade, speed, and time were gradually increased as the protocol progressed (Table 2).

#### 2.2.2. Interval Exercise

Interval exercise, a type of cyclic physical training that involves a series of high-intensity exercises (W stage) followed by a brief period of recovery or rest (resting stage), was provided three times a week for four weeks. It comprised 12 protocols, and each protocol consisted of four W stages (W1–W4) with high-intensity exercises and resting stages (R1–R5), including walking for active recovery. Each stage was carried out consecutively (i.e., R1 ⟶  W1 ⟶ R2 ⟶ W2 ⟶ R3 ⟶ W3 ⟶ R4 ⟶ W4 ⟶ R5 ⟶ finish). In protocols 1 to 6, the total exercise time was 2100 sec, and the exercise intensity was gradually increased as the protocol progressed. Protocols 7 to 12 lasted for 2160 sec, and the speed and slope were gradually increased as the protocol proceeded (Table 3).

### 2.3. Heart Rate

The HRs of two Beagles (CE1 and CE2) performing continuous exercise and two Beagles (IE1 and IE2) performing interval exercise were analyzed using a Polar H10 HR-monitor (Polar Electro Oy, Finland) throughout all exercises. After each protocol, the HR data were downloaded from the transmitters onto a computer using Polar Flow Software (Polar Electro Oy, Finland). The HR was reported as the mean values attained across the exercise stages.

### 2.4. Hematological and Serum Chemistry

Blood samples were collected from the jugular vein on the day before starting protocol 1, the day after protocol 6, and the day after protocol 12. Heparinized blood samples were centrifuged after blood withdrawal, and plasma was collected. In plasma, the biochemistry parameters (creatine kinase, CK; cholesterol, triglycerides, TG; and glucose) were measured using the Hitachi 7180 Auto analyzer (Tokyo Hitachi, Japan). In blood treated with ethylenediaminetetraacetic acid, the hematological parameters of white blood cells (WBC), red blood cells (RBC), hemoglobin (Hb), mean corpuscular hemoglobin (MCH), and mean corpuscular hemoglobin concentration (MCHC) were determined using ADVIA 2120i (Tarrytown, USA).

### 2.5. Data Analysis

For descriptive statistics, the comparison between the biochemistry parameters before exercise and after exercise was performed using paired *t*-tests. The HR differences between dogs in both exercise programs were assessed using repeated-measures analysis of variance. Post hoc tests were performed using the Bonferroni method. Pearson correlation calculations were used to determine correlations between individual HR and exercise intensity during continuous exercise or interval exercise. Statistical significance was set at *P* < 0.05 for all analyses. Statistical analysis was performed using GraphPad Prism 5.0 (San Diego, CA, USA).

## 3. Results

### 3.1. Heart Rate Response to Continuous Exercise

**Figure 1:**
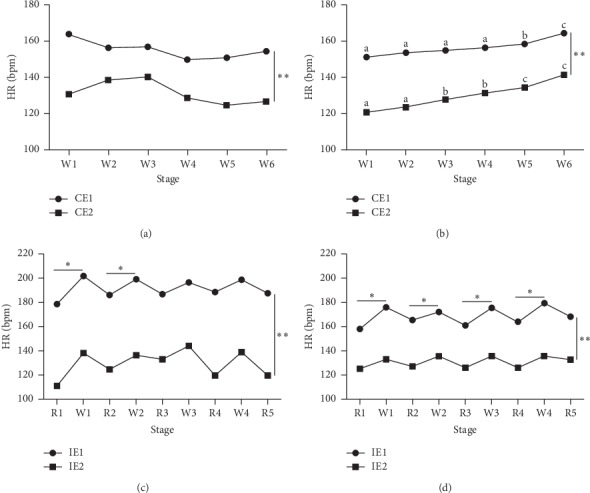
Mean heart rate values of each dog in continuous exercise in six W stages during protocols 1–6 (a) and protocols 7–12 (b). Mean heart rate values of each dog in the interval exercise in each R and W stages during protocols 1–6 (c) and protocols 7–12 (d). ^*∗*^^, a, b, c^Significant differences when comparing the average heart rate of each stage in an individual. ^*∗∗*^Significant differences (*P* < 0.05) in the mean heart rate between individuals at the same stage of the same protocol.

In the continuous exercise program, the HR results of the two dogs (CE1 and CE2) showed irregular responses during protocols 1 to 6, regardless of the change in exercise intensity, but showed an increasing trend with the increase in exercise intensity in protocols 7 to 12 (Figure 1(a)). The HRs of CE1 and CE2 were higher in W6 than in W1 (150 ± 11.5 beats/min (bpm) and 120 ± 10.1 bpm vs. 163 ± 13.8 bpm and 141 ± 3.0 bpm; Figure 1(b); *P* < 0.05), and the HR between the two Beagles in all protocols showed a significant difference (Figures 1(a) and 1(b); *P* < 0.05).

### 3.2. Heart Rate Response to Interval Exercise

The HRs in W1 and W2 of IE1 (199.8 ± 6.0 bpm and 199.6 ± 5.9 bpm, respectively) were significantly higher than that of R1 and R2 (176.4 ± 9.7 bpm and 184.4 ± 10.9 bpm; Figure 1(c); *P* < 0.05). The HRs of IE2 did not show a significant difference between both W and R stages in protocols 1 to 6 (Figure 1(c)). The protocols 7 to 12 were performed for 36 min with a gradual increase in exercise intensity, which was higher than that of protocols 1 to 6. In protocols 7 to 12, the HRs in W1, W2, W3, and W4 of IE1 (173.0 ± 14.8 bpm, 173.0 ± 12.9 bpm, 174.6 ± 12.0 bpm, and 178.8 ± 13.7 bpm, respectively) were higher than those of R1, R2, R3, and R4 (156.4 ± 15.2 bpm, 161.8 ± 11.21 bpm, 161.2 ± 12.4 bpm, and 165.2 ± 14.5 bpm, respectively; Figure 1(d); *P* < 0.01). Additionally, the HR of IE2 remained constant despite increased exercise intensity during protocols 7 to 12 (Figure 1(d)). The two Beagles also showed a significant difference in their HRs in every protocol (Figures 1(c) and 1(d); *P* < 0.05).

### 3.3. Correlation between Exercise Intensity and Heart Rate

In continuous exercise, the HR changes in CE1 and CE2 positively correlated with the exercise intensity during protocols 7 to 12 (Table 4(A); *P* < 0.05), but no correlation was observed during protocols 1 to 6. In interval exercise, exercise intensity and HRs of IE1 and IE2 were positively correlated in the whole protocol (Table 4(B); *P* < 0.05). Therefore, the HR of the dogs in all protocols of interval exercise was properly controlled according to exercise intensity changes, but the HR of dogs in the continuous exercise was controlled according to exercise intensity changes only in protocols 7 to 12.

### 3.4. Hematological and Serum Chemistry

The hematologic parameters of RBC, WBC, Hb, MCH, and MCHC of the four dogs were within the reference ranges both before and after exercise (Tables 5 and 6). For the four dogs performing continuous and interval exercises, CK significantly increased and cholesterol levels decreased. Although the values of TG and glucose decreased slightly, there was no statistical difference (Table 7).

## 4. Discussion

The purpose of this study was to develop, apply, and evaluate new continuous and interval exercise programs that followed the FITT-VP principle in dogs. In order to achieve our study goals, two types of exercise were applied, and their effects on HR and serum biochemistry parameters in Beagles were investigated. A few studies have reported that seizures may occur during exercise, indicating maladjustment or health problems [30, 31], but our results confirmed that the Beagles completed the continuous and interval exercise programs without any abnormal signs. To our knowledge, this is the first study to develop and evaluate training programs in dogs by applying the FITT-VP principle.

HR is the primary physiological determinant in the prediction of sudden cardiac death and general assessment of cardiovascular and metabolic disease progression [32, 33]. It is a useful tool to monitor cardiovascular fitness and to prevent overtraining and postexercise fatigue [34, 35]. In a dog, HR is one of the most critical parameters to assess the physical condition during exercise [22] and to determine cardiac output and maximal oxygen consumption levels, which are the typical indicators of aerobic capacity [36]. In the present study, HR was used as an indicator to verify the primary effect of continuous and interval exercise programs in Beagles. The average maximum HR in canines during progressive exercise testing reported in the previous study was 230 bpm [37], while in the current study, it was 223 bpm. The data indicated that the continuous and interval exercise programs are submaximal and may show a positive impact on cardiovascular fitness in all Beagles. In the continuous exercise protocols 1 to 6, the average HR at each stage of the protocols did not gradually increase with the intensity of the workout. The reasons why there is no association between the speed of activity and HR are not clear, but the lower and narrow range of exercise intensity may be involved. The treadmill speed in our protocols 1 to 6 was set at 4.8–6.4 km/h, which is similar to that of the study by Swanson et al. [38]. In this study, they demonstrated that the dogs were easily distracted at a lower treadmill speed, and the HR at a lower range of speed could hardly reflect exercise intensity [38].

In contrast, the average HR values of each stage showed a positive correlation with exercise intensity in the continuous exercise protocols 7 to 12 and all the interval exercise protocols. The data are consistent with the results of prior studies in which an increase in HR with an increase in the intensity of exercise was reported in humans and dogs [26, 39]. Another research group found that the HR in the second trial was significantly lower than that in the first trial when the test was repeated in dogs unfamiliar with treadmill exercise [23]. The study reported that repeated exercising decreases anxiety and increases learning effects, which could result in a decrease in HR. Nevertheless, we found that HR consistently responded to exercise intensity despite repeated experiments with 12 different protocols. These results suggest that the continuous exercise protocols 7 to 12 and all the interval exercise protocols are the recommended types of exercise to achieve the purpose of cardiovascular fitness training.

Changes in HR due to exercise are made through complex mechanisms of the autonomic nervous system, hormones, circulation system, and cardiovascular metabolism [40]. HR is increased by stimulating the contraction of the heart with the activity of the sympathetic nerve and suppression of the parasympathetic nerve during exercises that increase the energy demand [41]. Moreover, the HR response reflects increased cardiovascular stress and strain and enhanced respiratory oxygen uptake in response to higher physiological demand [26, 39], enhances cardiac contractility, coronary perfusion, and vascular formation, and reduces myocardial infarction and oxidative stress [42]. Based on this background, it is believed that the changes in HR identified during exercise in this study could have had a positive impact on the cardiovascular fitness of the Beagles.

Hematological parameters were evaluated to investigate the effects of two different exercise programs in Beagles. Elevation of RBCs and WBCs during exercise is associated with stress or inflammation [43]. In this study, the RBCs and WBCs of all Beagles were within normal ranges before exercise, two weeks after exercise, and four weeks after exercise (data at two weeks after exercise were not shown). Previous studies have reported that high-intensity exercises reduce Hb levels due to the release of bone marrow hematopoietic stem cells [44]. Moreover, MCH and MCHC levels significantly decreased after excessive exercise, which may lead to hypochromic anemia [45]. However, the data of the present study indicated that Hb, MCH, and MCHC of all Beagles were within reference ranges and remained almost the same after exercise. Thus, we believe that the two exercise programs applied to this study are suitable for dogs. Unlike the hematological results, the CK levels were altered after continuous and interval exercises in Beagles. The increased serum CK after exercise suggests minor musculoskeletal damage [46]. The results are congruent with previous findings of other studies [47, 48]. These outcomes may be influenced by free radicals produced during exercise that cause changes in the permeability of muscle cell membranes, resulting in increased CK, which are the biomarkers of muscle damage [49]. Another contributing mechanism could be the involvement of an increase in blood urea due to an overload of amino-acid metabolism in the liver and muscles [50]. However, the CK levels returned to the normal range after finishing the exercise program, indicating that the increase could have been a transient phenomenon due to exercise [47].

Plasma glucose is an important energy source for contracting skeletal muscles [51]. Skeletal muscle extracts glucose from the blood to maintain the demand for carbohydrates as an energy source during exercise [52]. During prolonged exercise, plasma glucose levels drop due to a reduction in hepatic glucose production [53]. In humans, continuous exercise leads to many positive metabolic effects, such as decreased blood glucose levels and reduced body fat [54]. However, the data in this study did not show any change in glucose levels after exercise. The mechanisms involved have still not been elucidated, but the results could vary due to some influencing factors, such as exercise protocols, exercise training periods, and time points of measurement. Hypercholesterolemia is also a significant risk factor for cardiovascular diseases [55]. Previous studies in humans demonstrated that aerobic exercise lowered cholesterol levels, thus improving the cardiovascular system and reducing the risk of coronary heart disease and stroke [56, 57]. In dogs, hypercholesterolemia also increases the risk of cardiovascular disease, arteriosclerosis, stroke, and metabolic syndrome [58–60]. Various drugs are being developed and used to treat this in veterinary medicine [61]. Interestingly, we confirmed that the continuous and interval exercises effectively reduced cholesterol levels in postexercise Beagles. Thus, the exercise programs developed in this study could be used as an alternative method to prevent cardiovascular and metabolic diseases. While continuous and interval exercises significantly decreased cholesterol levels, they had only few positive benefits on TG, which was incongruent with previous findings [62]. According to previous research studies in mice and humans, participants with low baseline TG levels showed a slight decrease after exercise [63], whereas high TG levels showed a substantial reduction after exercise [62]. Thus, the different TG responses to exercises could be dependent on the initial TG levels of the individuals.

In conclusion, the continuous and interval (especially protocols 7 to 12) exercise programs developed in this study induced an appropriate HR reaction in Beagles with no adverse effects on the hematological parameters. Although a small number of samples were used in our study, we found significant changes in serum parameters and HRs, which reflected the potential positive effects of continuous and interval exercises on physical fitness, performance, and health in dogs. Because the continuous and interval exercise programs do not exhibit any harmful effects, further studies need to be conducted to evaluate the physiological function, genetic interactions, and cardiovascular fitness.

## Figures and Tables

**Table 1:** Baseline Beagle's information by group.

Group	Name	Age	Sex	Weight (kg)
Continuous	CE1	2 years	Male	9.8
	CE2			6.5

Interval	IE1	2 years	Male	7.7
	IE2			7.1

CE1 and CE2 are Beagles that performed continuous exercise. IE1 and IE2 are Beagles that performed interval exercise.

**Table 2:** Continuous exercise program consisting of 12 protocols.

Protocol	Divide	W1	W2	W3	W4	W5	W6	Duration of exercise
1	Sec	300	300	300	300	300	300	1,800
	Grade	0	0	0	0	0	0	
	km/h	4.8	4.8	4.8	4.8	4.8	4.8	

2	Sec	400	400	400	400	400	400	2,400
	Grade	4	4	4	4	4	4	
	km/h	4.8	4.8	4.8	4.8	4.8	4.8	

3	Sec	400	400	400	400	400	400	2,400
	Grade	4	4	4	4	4	4	
	km/h	5.6	5.6	5.6	5.6	5.6	5.6	

4	Sec	500	500	500	500	500	500	3,000
	Grade	6	6	6	6	6	6	
	km/h	5.6	5.6	5.6	5.6	5.6	5.6	

5	Sec	500	500	500	500	500	500	3,000
	Grade	6	6	6	6	6	6	
	km/h	6.4	6.4	6.4	6.4	6.4	6.4	

6	Sec	600	600	600	600	600	600	3,600
	Grade	6	6	6	6	6	6	
	km/h	6.4	6.4	6.4	6.4	6.4	6.4	

7	Sec	600	600	600	600	600	600	3,600
	Grade	0	2	4	6	8	10	
	km/h	5.2	5.6	6	6.4	6.8	7.2	

8	Sec	600	600	600	600	600	600	3,600
	Grade	5	6	7	8	9	10	
	km/h	5.2	5.6	6	6.4	6.8	7.2	

9	Sec	600	600	600	600	600	600	3,600
	Grade	2	4	6	8	12	12	
	km/h	5.4	5.8	6.2	6.6	7	7.4	

10	Sec	600	600	600	600	600	600	3,600
	Grade	7	8	9	10	11	12	
	km/h	5.4	5.8	6.2	6.6	7	7.4	

11	Sec	720	720	720	720	720	720	4,320
	Grade	4	6	8	10	12	14	
	km/h	5.6	6	6.4	6.8	7.2	7.6	

12	Sec	840	840	840	840	840	840	5,040
	Grade	9	10	11	12	13	14	
	km/h	5.6	6	6.4	6.8	7.2	7.6	

**Table 3:** Interval exercise program consisting of 12 protocols.

Protocol	Divide	R1	W1	R2	W2	R3	W3	R4	W4	R5	Duration of exercise
1	Sec	240	180	300	180	300	180	300	180	240	2,100
	Grade	0	1	0	2	0	3	0	4	0	
	km/h	4.8	5.8	4.8	6	4.8	6.2	4.8	7.2	4.8	

2	Sec	240	180	300	180	300	180	300	180	240	2,100
	Grade	0	3	0	4	0	5	0	6	0	
	km/h	4.8	6	4.8	6.2	4.8	6.4	4.8	7.2	4.8	

3	Sec	240	180	300	180	300	180	300	180	240	2,100
	Grade	0	5	0	6	0	7	0	8	0	
	km/h	4.8	6.2	4.8	6.4	4.8	6.6	4.8	7.2	4.8	

4	Sec	240	180	300	180	300	180	300	180	240	2,100
	Grade	0	7	0	8	0	9	0	10	0	
	km/h	4.8	6.4	4.8	6.6	4.8	6.8	4.8	7.2	4.8	

5	Sec	240	180	300	180	300	180	300	180	240	2,100
	Grade	0	9	0	10	0	11	0	12	0	
	km/h	4.8	6.6	4.8	6.8	4.8	7	4.8	7.2	4.8	

6	Sec	240	180	300	180	300	180	300	180	240	2,100
	Grade	0	11	0	12	0	13	0	14	0	
	km/h	4.8	6.8	4.8	7	4.8	7.2	4.8	7.2	4.8	

7	Sec	180	180	360	180	360	180	360	180	180	2,160
	Grade	0	7	0	8	0	9	0	10	0	
	km/h	4.8	7.4	4.8	7.6	4.8	7.8	4.8	8	4.8	

8	Sec	180	180	360	180	360	180	360	180	180	2,160
	Grade	0	9	0	10	0	11	0	12	0	
	km/h	4.8	7.4	4.8	7.6	4.8	7.8	4.8	8	4.8	

9	Sec	180	180	360	180	360	180	360	180	180	2,160
	Grade	0	11	0	12	0	13	0	14	0	
	km/h	5.2	7.4	5.2	7.6	5.2	7.8	5.2	8	5.2	

10	Sec	180	180	360	180	360	180	360	180	180	2,160
	Grade	0	11	0	12	0	13	0	14	0	
	km/h	5.4	7.4	5.4	7.6	5.4	7.8	5.4	8	5.4	

11	Sec	180	180	360	180	360	180	360	180	180	2,160
	Grade	2	11	2	12	2	13	2	14	2	
	km/h	5.6	7.6	5.6	7.8	5.6	8	5.6	8.2	5.6	

12	Sec	180	180	360	180	360	180	360	180	180	2,160
	Grade	4	11	4	12	4	13	4	14	0	
	km/h	5.8	7.8	5.8	8	5.8	8.2	5.8	8.4	5.8	

**Table 4:** Correlation between individual heart rate and exercise intensity during continuous exercise or interval exercise.

	CE1-A	CE2-A	CE1-B	CE2-B
(A)				
CE1-A				
CE2-A	0.393			
CE1-B	−0.571	−0.552		
CE2-B	−0.661	−0.608	0.984^*∗*^	

	IE1-A	IE2-A	IE1-B	IE2-B
(B)				
IE1-A				
IE2-A	0.863^*∗*^			
IE1-B	0.922^*∗*^	0.796^*∗*^		
IE2-B	0.837^*∗*^	0.730^*∗*^	0.916^*∗*^	

A is the correlation between individual heart rate and exercise intensity during continuous exercise. B is the correlation between individual heart rate and exercise intensity during interval exercise. CE1-A means the average heart rate of a CE1 dog during protocols 1–6 of continuous exercise. CE1-B means the average heart rate of a CE2 dog during protocols 7–12 of continuous exercise. IE1-A means the average heart rate of an IE1 dog during protocols 1–6 of interval exercise. IE1-B means the average heart rate of an IE2 dog during protocols 7–12 of interval exercise. ^*∗*^Significant correlations (*P* < 0.05).

**Table 5:** Changes in hematological parameters after continuous exercise in dogs.

Parameters (unit)	Before exercise	After exercise	Before exercise	After exercise
	CE1		CE2	
RBC (M/*μ*l)	6.6	6.5	6.8	5.0
WBC (K/*μ*l)	7.4	9.6	5.4	3.7
Hb (g/dl)	15.7	15.2	12.5	11.8
MCH (p/g)	23.8	23.5	23.3	23.7
MCHC (g/dl)	36.4	36.0	36.1	34.7

CE1 and CE2 are Beagles that performed continuous exercise.

**Table 6:** Changes in hematological parameters after interval exercise in dogs.

Parameters (unit)	Before exercise	After exercise	Before exercise	After exercise
	IE1		IE2	
RBC (M/*μ*l)	6.9	6.5	5.2	5.4
WBC (K/*μ*l)	7.0	5.6	6.4	5.4
Hb (g/dl)	16.8	15.9	12.1	12.4
MCH (p/g)	24.6	24.4	23.2	22.8
MCHC (g/dl)	36.4	35.5	35.7	34.8

IE1 and IE2 are Beagles that performed interval exercise.

**Table 7:** Changes in biochemistry parameters in four Beagles subjected to continuous or interval exercise.

Parameters (unit)	Before exercise	After exercise
	(Mean ± SD)	(Mean ± SD)
Creatine kinase (U/L)	108.8 ± 23.3	221.8 ± 75.1^*∗*^
Cholesterol (mmol/L)	146.8 ± 21.5	124.8 ± 18.3^*∗*^
Triglycerides (mmol/L)	45.3 ± 17.0	33.3 ± 3.9
Glucose (mg/dL)	113.5 ± 14.8	97.0 ± 13.9

^*∗*^Significant difference between before and after exercise (*P* < 0.05).

## Data Availability

All data used to support the findings of this study are included within the article. The analyzed data during the current study are available from the corresponding author upon reasonable request.
